# Exclusion Through Inclusion. Struggles Over the Scalar Regimes of Belonging *Europe* and the *Family* at the 1995 Fourth UN World Conference on Women and the Agency of *(Polish) Women*

**DOI:** 10.3389/fsoc.2019.00055

**Published:** 2019-07-16

**Authors:** Jennifer Ramme

**Affiliations:** ^1^Polish-German Cultural-and Literary Relations and Gender Studies, Faculty of Cultural and Social Science, European University Viadrina, Frankfurt, Germany; ^2^The Viadrina Institut für European Studies, Frankfurt, Germany

**Keywords:** scales, agency, familism, transnational feminism, Poland, Central and Eastern Europe (CEE), politics of belonging, United Nations (UN)

## Abstract

Gender regimes of belonging are contextually variable and closely linked to other regimes of belonging, such as the *family*, the *nation*, or, the *region*. In the case of Poland, this contextuality and interdependence becomes apparent when analyzing struggles between feminist and Catholic anti-choice environments. While the first group opts for gender democracy, the other favors a familistic social order. In the mid-90s, the struggle over “Polish” gender regimes took an international dimension and was played out as well at the international fora of the UN. When women's rights actors from Poland appeared at the 4th World Conference on Women held by the UN in China in 1995, they experienced being positioned within a pre-structured spatial order and learned that this positioning and the synthesis within scalar regimes of belonging, such as the scale of *family* but also that of *region*, have a major impact on their political agency. The spatial order of the UN is a field of conflict, as certain positioning within regimes of belonging might limit political agency and therefore constrain the making of claims. NGOs struggle to get representation and definitional power over space and collective identity categories, but they also put effort into changing the very composition and hierarchies within identity regimes. Toward this end, they form coalitions and networks and perform group identities and may even act in opposition to institutionalized regimes of belonging. The use of concepts such as *belonging* and *scale* allows us to avoid the analytical limits that are linked to the theoretical frameworks of recognition and identity politics. This article explores the strategies of scalar politics of belonging applied by NGOs, which also lead to the establishment of bodies representing women from the post-socialist countries of Central and Eastern Europe, such as the Karat Coalition, but it also draws attention to the political effects of scaling belonging through the “family” or “Europe.” Today, the question as to what shape gender regimes of belonging should take is still an important site of struggle.

## Introduction

This article looks at the importance of scales in struggles to obtain political agency and representation in and through spatial and scalar constructions of belonging, such as *European women* or the *Polish family*. Firstly, I am concerned with representation, definitional power, and agency through scalar constructions, and secondly, with the other- and self-positioning of social actors in hierarchical regimes of belonging. In examining the strategies of feminist actors from Poland to attain political agency through spatial and identity constructs, I take the example of struggles over gender regimes in Poland and a United Nations meeting and then consider the importance of *scale* in the development of regional advocacy organizations for gender democracy and in struggles between familistic Catholic and gender-democratic actors. *Scale* is understood by me as a formal and sensible organizing principle that describes the integration of units into other subordinate units. The very concept of *scale* is helpful for analyzing the organization of regimes of belonging, their intertwining with spatial constructs and the possible effects *scaling* can have on political agency.

I concentrate firstly on why the regional scale is chosen (beyond the national framework) in the collective self-positioning of non-governmental actors and organizations, and secondly, on the possibilities available in the context for acting as a collective subject to formulate political demands from this position. The social and political context is provided by United Nations processes which produce interactional spaces that include so-called “civil society” actors^1^. I address the relational constructions of “regions” and “identity” and their scaling with regard to the external categorization and the self-definition of emerging collective subjects. This process is accompanied by boundary drawing, by the organization of inclusion and exclusion, and by the definition of relations in a spatial order of belonging.

My argument is 2 fold. Firstly, taking a perspective on scalar dimensions of organizing belonging allows me to analyze struggles which are often portrayed as “identity politics” or “struggles for recognition,” beyond the most common frameworks of identity and recognition. Secondly, my aim is to draw attention to the political dimension of hierarchically organized scalar constructs of belonging, the effects the scaling of belonging have on political agency, and finally, the similarity between certain scalar formations.

The organizations and actors under study have to do with feminist and gender democracy initiatives that arose in Poland largely in the late 1980s and early 1990s^2^. System change, which involved the final surrender by the Polish United Workers' Party (Polska Zjednoczona Partia Robotnicza, PZPR) in 1989 of its monopoly on power, pluralized the political landscape further. It also mobilized many actors at home and abroad intent on influencing the future shape of government and society in Poland. How the topic of abortion was addressed provides one example of this pluralization. In Poland, the subject provoked heated public discussion (see, e.g., Chałubiński, 1994; Fuszara, 1994; Chołuj and Neusüß, 2004a) that has continued to this day. In 1989, a bill prohibiting abortion was tabled and debated by the Senate (second chamber of parliament). It was based on a draft prepared by episcopate staff (Jankowska, 1991; Ignaciuk, 2007). However, the Catholic Church had first sought to influence the law on this subject in the late 1980s. After 1989, the legality of abortion and women's rights in general were debated in the context of whether Poland was to adopt a laicist or Catholic orientation. Despite numerous protests, religion was introduced in schools and the ban on abortion was passed in 1993, which still applies today.

From the very first, the future shape of the Polish state was discussed far beyond the borders of the country, too, involving numerous international and transnational actors (e.g., the Catholic Church, World Bank, European Union, United Nations, particularly the United Nations Development Programme), but also private donors, NGOs, and religious movements from all over the world. Opportunities were thus seized to influence the development of Poland through advocacy in international forums. The 1995, the Fourth UN World Conference on Women was not only a crucial event, a deciding moment within the struggles over “Polish” gender regimes, but also deserves attention, as analyzing this particular event allows us in many ways to unfold the complicated nature of those struggles, elements of which can still be observed today.

The processes and meetings organized by the UN Economic and Social Council on the subject of women's rights brought together both government and civil-society actors of various political persuasions, establishing important intermediary spaces (prior to accession to the European Union) where the shape of the Polish state was negotiated. This context of action played a key role in regional alliance building and in the development of Central and Eastern European and Central Asian NGOs. Both the contexts of action and the constitution of collective subjects (for example, the Karat Coalition), in regard to scalar dimensions of familistic^3^ and European regimes of belonging, their analogy and their impact on political agency, have hitherto attracted little scholarly attention. That scale matters and that scaling belonging is of crucial importance for successful politics, not only in the 1990s but today as well, can be observed in, for example, the intensified activities of international right-wing familist alliances, that work to change not only national but the UN human rights frameworks as well, in such a way that would broaden the playing field for the conservative, religious and (far) right.

## Materials and Methods

The article presents outcomes from research focusing on the struggles of “Polish” gender regimes and the reasons behind the development of regional organizations that represent women from former state-socialist Europe. This research was done in the frame of a broader international research project on the “Transnationalization of Struggles for Recognition.” The research was conducted with mixed methods^4^. I took a sample of initiatives and actors that have been involved in UN-processes, that have “polish” origin and were involved in struggles about the composition of “Polish gender regimes,” who organized themselves regionally or/and beyond nation states. They include, above all, SKOP—‘95 (Społeczny Komitet na Rzecz Organizacji Pozarza̧dowych Pekin 1995, Polish Committee of NGO's—Beijing 1995'), the Karat Coalition, and the Central and Eastern European Women's Network for Sexual and Reproductive Health and Rights (ASTRA). Other organizations looked at in this context are regional and transnational networks, such as the Network of East-West Women (NEWW), as well as local women's organizations in Poland that have defended women's rights outside the national framework, too. My sources include, among others, documents from the archives and websites of the NGOs under study^5^, as well as United Nations documents and press releases. I also conducted in-depth qualitative interviews with actors and experts who had participated in the processes and events under study^6^. The interviews helped me to identify what moments and events were seen as crucial in regard to transnational feminist activism in Poland and its impact. A further method employed was participant observation between 2009 and 2013 and in 2017 at conferences, workshops, and internal meetings of organizations^7^.

An important finding during my initial research was that there still exists no definition neither of “the region” nor on its scope and borders (beyond the state-socialist past) that would be shared by all actors and initiatives under study. The analysis, therefore, focused on how institutionalized scalar regimes of belonging (such as the region at the UN or the family institution) are negotiated, contested and might contextually change in terms of their form (composition, scaling, hierarchies).

I integrate research perspectives from critical cultural studies, gender studies and queer theory, the arts, political aesthetic theory, and political and human geography. The theoretical framework applied within this analysis is transdisciplinary, with a strong emphasis on thinking in terms of critical aesthetic theory (Rancière, 2006) and processual spatial theory (Löw, 2001). This research perspective moves the practice division and the ordering of “the sensible” (Rancière, 2006) in politics of gender-democratic actors and their opponents to the center of the analysis. The methodological approach is combining elements of critical discourse analysis Jäger, 2015, an analysis of “divisions of the sensible” (Rancière, 2006) and processual productions of space (Löw, 2001) in the practice and discourses of the actors under study. I also applied methods of close observation and reconstruction of social actions next to their context and provide subjective explanations of the actors involved.

## Concepts and Research Perspectives: Hierarchies of Scale and Belonging

It's no surprise that research on scales and the discussion of *scale* as an analytic concept is most advanced in the discipline of geography, since defining territories and spaces as objects of study is crucial for research in this field. As a consequence, the critical discussion of certain normative or essentialist assumptions within the discipline (for example, about the “scale”) are objects of lively dispute, which disciplinary outsiders can also learn from. Critical human geographers, including feminists, question the scholarly use of spatial scales as hierarchizing and ahistorical units (see, e.g., Marston et al., 2005)^8^. For instance, they problematize the hierarchizing effect of scaling in such terms as micro, meso, macro; national, international, transnational; space, place; and local, global. Scaling of this sort treats the global as abstract and is independent of the local and concrete (Massey, 2002, 2004). The bird's-eye view that the scalar perspective provides of the world from a supposedly objective position marks a further dichotomy between universal and particular. Doreen Massey suggests treating space instead in terms of relations, paths, connections, and understanding the global as being concretely located (Massey, 2002).

This criticism has induced some scholars to favor abandoning all use of scale to avoid reinforcing its discursive and naturalizing effects (Marston, 2000; Marston et al., 2005). The concept of horizontal networks is proposed to replace vertical and hybrid theories of space (Marston et al., 2005).

Legg, however, considers the idea of completely rejecting scale as a vain attempt to obviate the effects of scalar practices. “The unequal distribution of power relations in the world make a human geography without scale both idealist and unrepresentative of the lived and historically specific hierarchies, which scalar rhetoric and technologies create, both epistemologically and ontologically” Legg (2009). “Scale,” as other authors argued before, “is not simply an external fact awaiting discovery but a way of framing conceptions of reality” (Delaney and Leitner, 1997, p. 94–5). Similar arguments on “regional identity” have been brought up by the sociologist Pierre Bourdieu, who writes that such classifications as “regions” are based on “characteristics which are not in the slightest respect natural and are to a great extent the product of an arbitrary imposition, in other words, of a previous state of the relations of power in the field of struggle over legitimate delimitation” (Bourdieu, 1991, p. 223). Since *scale* is a way to frame reality, one could argue that we do not need the term *scale*, because there exists the term *frame*. Fraser has this to say about frame-setting, which she illustrates with the division of political space within the Westphalian Order: “Far from being of marginal significance, frame-setting is among the most consequential of political decisions. Constituting both members and non-members in a single stroke, this decision effectively excludes the latter from the universe of those entitled to consideration within the community in matters of distribution, recognition, and ordinary representation” (Fraser, 2009, p. 19). While Fraser's observation is also relevant for the research presented here, I decided to use the concepts spatial order, belonging, and scale because the concepts frame and frame-setting do not adequately cover relations within and outside of a frame or the possible relations and multiplicity of structures that order belonging.

Hierarchizing spatial narrations can be converted into powerful spatial orders. Scales are not only imaginary in a symbolic and spatial sense but, due to the hierarchies and bordering effects they produce, also form a lived reality for individuals. Bodies move in spaces that are created by human agency and/or defined in the course of social exchanges. They repeatedly come up against the limits of materialized spatial orders, but also participate themselves in constituting such arrangements. Spatial arrangements, as described by Löw (2001), are embedded in institutions and secured through resources.

The production of scales is tied to power. Erik Swyngedouw explains this as a process: “I conceive scalar configurations as the outcome of sociospatial processes that regulate and organize power relations. Scale configurations change as power shifts, both in terms of their nesting and interrelations and in terms of their spatial extent. In the process, new significant social and ecological scales are constructed, others disappear or become transformed” (Swyngedouw, 2004, p. 132–3, quoted from Marston et al., 2005, p. 418).

Thus, space not only takes the form of equal relations but also appears as space within space, as space that encompasses other spaces or ties them together. Scale can therefore be, among others, understood as a form of organizing space, which is capable of producing hierarchical effects by enclosing a smaller unit within a larger one.

Theorists of scale argue that scale does not necessarily need to take the shape of a vertical top-down hierarchy (Leitner et al., 2008, p. 161), nor that the smaller unit is always dominated by the larger one (Collinge, 1999). In the case of dominant hierarchizing integration through scalar practices, exclusion might be achieved through inclusion. In the case of dominant scalar subordination, the unit subordinated by scale can exist only in relation to the superordinate unit. In a political situation, such subordination could imply that a unit is not fully recognized as independent or prevents its performing as an independent unit.

I use the term scale not only with reference to geographical ordering units. I understand scaling rather as an ordering principle that can be applied to various formations of spatial order, but not to these alone. This includes spatial allocations in the process of subject constitution as well as the subordination of people by their integration in identitary constructions and the attribution of social roles. The relationship between scale and identity, gender and sexuality, in regard to regional representation and geopolitics, has also been recognized by other scholars, although mainly from the field of political and human geography (e.g., for more recent publications Conway, 2008; Binnie, 2016). Many geographers, especially feminist ones, argue that scaling processes also include smaller units than for example states or regions, such as the body or the household/home (Smith, 1992, 1996; Marston and Smith, 2001). However, my understanding of the term *scale* is not limited to a (human) geographical understanding. I attempt instead to integrate non-essentialist critical perspectives inspired above all by political aesthetics in the tradition of Jacques Rancière, in particular his theory on the “division of the sensible,” which draws attention to the politics of the division of space, time, and identities, as well as their intersections Rancière (2006). I therefore understand scales as a specific way of arranging, bordering, and dividing “the sensible” that often implies specific divisions of time, space, and identities.

In order to grasp the entanglement of space, territory, and identity-categorization within regimes of belonging, I have also borrowed the concept of *(An)Ordnung*. That concept originally refers to *space* only and was introduced by Martina Löw in her “Sociology of Space” in 2001, where she is combining the terms *Anordnung* (positioning toward something) and *Ordnung* (order). According to the sociologist, this synthesis and the relational positioning, arrangement/ordering [*(An)Ordnung*] of living beings and material can be undertaken by the subjects themselves or by outsiders (Löw, 2001). It indicates that the process of producing space involves an act of arranging and the production of order. In my view, the perspectives applied within critical theories of space combined with perspectives coming from political aesthetic theory are suitable for analyzing the constitution of subjects and processes of identity construction. When I use the term “identity” I am referring to a category of practice and an outcome of human categorization and socio-political processes that also defines the position of the subject in relation to other positionings. The subject (representing an “identity”) is “identified” and synthesized with other subjects and/or material to form shared regimes of belonging, which have spatial dimensions as well.

A very popular and inspiring but also slightly different understanding of the concept of *belonging* is that which Nira Yuval-Davis introduced in her book *The Politics of Belonging: Intersectional Contestations* in 2011. Yuval-Davis links belonging to social locations and emotional attachments, to the identifications and identities of individuals and their attachments to collectivities and groupings (2011). The term *belonging*, as used in this article, is meant as a formalist and aesthetic category and stands in opposition to the notion of “identity.” *Belonging* is an analytical term for describing relational organizing principles capable of arranging “the sensible” (as understood by Rancière, 2006) and forming attachments among its elements (such as, for example, “identities” and “territories”), while *scale* refers to the hierarchies and boundaries.

Use of the concepts *scale* and *regimes of belonging* and their understanding as particular orders of “the sensible” allows us to capture the practices of the organizations under study and the associated processes with greater precision. Furthermore, the essentialization of “identity” constructions can be more likely avoided. This approach makes it possible to show not only the processes of constructing scales but also the situatedness, relativity, and, above all, changes in subject positionings and constructions of “identity,” “gender” and “region.”

## The Importance of Scale and Belonging in Feminist Struggles. The Debate on Space and Representation at the Fourth World Conference on Women of the United Nations in 1995

Taking the example of the 1995 Fourth World Conference on Women of the United Nations in Beijing and the parallel NGO summit in Huairou in China, I examine inclusion and exclusion through the scalar construction of space, territory and identity.

Among the feminist actors from Poland who went to Beijing were members of SKOP. The SKOP initiative had been organized in 1994 by NGO representatives and individuals in Poland to prepare a “shadow report”^9^ for the Fourth World Conference on Women (Fuchs, 2003; Chołuj and Neusüß, 2004a)^10^. At the preparatory regional conference for the World Women's Conference, in Vienna in 1994, which was open to NGOs from Europe and North America, there was a small group of women from Eastern European NGOs. In the view of the NGO activists from Poland who were present and that I have interviewed, the draft report of the Polish government prepared in 1994 presented the situation of women in Poland incorrectly^11^. Aleksandra Solik from the SKOP—‘95 initiative explained to me, that the report was “full of gender stereotypes and indicated a complete misunderstanding of the essence of women's rights as well as the government commitments in the area of gender equality policy”^12^. According to Chołuj and Neusüß (2004a), the new post-state socialist liberal government had moved closer to the Catholic Church and submitted to the pressure of the Church in order to obtain support for EU accession. The government's report stated, for example, that female unemployment resulted from women's overactivity (Chołuj and Neusüß, 2004a, p. 184). On the basis of experience with successful advocacy work in Vienna (1994 preparatory regional conference for the Fourth World Conference on Women in 1995), the local NGO actors decided to mobilize and unify women's rights activists in Poland in preparation for the Fourth UN World Conference on Women. This was the origin of SKOP—‘95. Under pressure from the NGO advocacy in Vienna, the Polish government revised their report.

The feminist actors who had organized themselves to strengthen women's rights in Poland and address demands to the Polish government found a new field of activity for their political struggles in the forums of the United Nations. The Fourth World Conference on Women and the preparatory processes thus reorganized relations between states and civil society as promoted by the United Nations (in this case women's rights activists). The conference enabled actors to assume representational functions (a subject position that had previously been reserved to the state).

For actors from Poland, however, there were two further changes in their own subject position within scalar inclusion and exclusion practices: first, recognition of the autonomy of the category woman and introduction of the category gender, and, second, classification in the category Europe.

### Recognizing the Autonomy of the Category Woman in the Context of Poland

In the 1990s, after the disintegration of the political Eastern Bloc and Soviet Union, shifts occurred in global orderings and power relations. System change in Poland led to the reorganization of the state and society. Debates on the shape the new Poland would take addressed, among other things, the category woman and its subordination to the category family. The topic of reproductive rights and, later, criticism of domestic violence, which national Catholic actors took to be an attack on the integrity of the family, were and have remained focal themes in these debates^13^.

In matters of representation, the struggle to establish the office of a national women's commissioner in Poland was a telling example of this, as the frequent changes in the title of the authority show. As power changed hands, the word woman was replaced by family. After the Third World Conference on Women in Nairobi in 1986, the office was set up by the communist government under the name “Government Commissioner for Women.” In 1991, after a coalition of center-right and national-Catholic parties (under Krzysztof Bielecki) came to power, the name was changed to “Commissioner for Women and Family.” In 1995, it was renamed “Commissioner for Family and Women” (1995^14^−1997), and then merely “Commissioner for Family”^15^. From 2001 to 2005, the authority was titled “Government Commissioner for the Equal Status of Man and Woman” only to be abolished until resuscitated in 2008 under pressure from NGOs and the EU under the name “Government Commissioner for Equal Treatment,” avoiding any mention of who was to be treated equally.

In the national-Catholic view, espoused by, among others, parties close to the Catholic Church the category woman functions always in relation to the categories man/husband and family^16^. This view is shared by the Polish and the international Catholic Church (e.g., Raday, 2012a). In contrast to this, feminist actors fight for the autonomy of the category woman, to allow it to function in a range of relations and to give agency to the people placed in this category. In this context, agency means the freedom to order/arrange oneself relationally and also to represent oneself. The relational and complementary tying of woman to man (with man being treated as universal and, unlike woman, able to function autonomously) is seen by feminist actors and feminist theory as a discriminatory relation of dominance (e.g., Beauvoir, 1989; Butler, 1991).

For feminist activists from Poland, the United Nations provided a political opportunity. Thus, Human Rights law extended the right to equality to all family members thus “repudiating patriarchal hierarchy in the family” (Raday, 2012a, p. 227)^17^.

The recognition of the autonomy of the category woman and of women's rights as an integral part of human rights (see the Vienna Declaration of the 1993 World Human Rights Conference), as well as the goal of the 1979 UN CEDAW^18^ convention and the 1995 Beijing Platform for Action to eliminate discrimination against women therefore changed the subject positions^19^. It enabled women's rights activists to assume spokesperson and representational functions. Activists were enabled to “occupy” the category woman, a position from which they could then negotiate and address demands to the Polish government. By contrast, political representatives of conservative Catholic actors in Beijing and Huairou found their scope for action weakened and the representational position already occupied. This had to do with, among other things, the circumstance that Catholic, anti-choice actors had underestimated the importance of the forum and had decided to participate only at a very late date (compare Fuchs, 2003, p. 127). Women's organizations struggling for women's rights had been involved much earlier in the Beijing and UN processes, while Catholic women did position themselves in opposition to the forum by contradicting its basis. They thus stated in the opening sentence of the shadow report that women in Poland were not discriminated against. For they positioned themselves in both debates and in the shadow report not only as “Polish women” but also as members of “Polish families.” In the report, the organizations advocated, for example, restriction of the right to divorce (opposing divorce “on demand”). Research describing the developments at the UN World Conference on Women in Beijing and analyzing the reports and demands has pointed out the differences between the shadow reports, one provided by the gender-democratic NGO organized within SKOP—‘95 and the other prepared in response by national-Catholic groups (e.g., Fuchs, 2003, 2004, p. 48; Fuszara, 2005). The final postulate of the report relates only to families and mothers (Polska Federacja Ruchów Obrony Życia, 1995). The women who represented the Catholic pro-life and family organizations from the Polish Federation of the Defense of Life (Polska Federacja Ruchów Obrony Życia, 1995) were in a contradictory situation when they positioned themselves solely as women. They were on the defensive when, with respect to an autonomous understanding of the category woman, they repeatedly had to point to the relational integration of women in the superordinate scale of family.

### Classification in the Category Europe. Regional Spatial Orders and Strategies for Interest Representation

Women's rights activists at the conference in 1995 experienced not only scalar autonomy. In the United Nations, the general category woman is produced through positioning in a regional order by integrating state territories and state areas of authority. With regard to generalization in the UN context, this means that the subjects of civil society are ordered as follows: human beings of the world, women and human beings, women in general (globally), women of a region, women of a state.

Lohmann of the SKOP—‘95 initiative and later co-founder of the Karat Coalition notes: “Our presence in Beijing made us aware of how important it was to belong to a region” Lohmann (1997, p. 27).

Not only the consultation processes for the Beijing Declaration and the Beijing Platform for Action but also the strategically important NGO Caucuses at the conference were organized on a regional pattern.

The United Nations (UN Economic and Social Commission) divides the world into five regions^20^. This division and the allocation of seats are guided by geopolitical and economic categories and not by purely geographical criteria. The division into regions is decided on the basis of, among other things, common political and cultural heritage, religion, development status, economic relations, and only partly in terms of geographical location^21^. What is more, the countries to be included and the names of regions and subregions change, so that multiple memberships occur^22^.

In the Beijing Process, the principle of regional division took over, which meant that separate regional consultations were organized and regional papers were prepared. The situation is different under the Convention on the Elimination of All Forms of Discrimination against Women (CEDAW), which requires states to report only individually. The result is that work on CEDAW shadow reports are submitted at different times by member countries. The situation is similar for NGO mobilization.

Being organized on a regional basis, the Beijing process accordingly allowed the regional presence and consolidation of NGOs and civil society actors. NGO-specific spatial orders reflected the spatial order devised by the United Nations, since the institutionalized and administrative spatial orders influenced the networking and consolidation of NGOs, which were also assigned to regions and were addressing UN processes.

For women's rights NGOs, accepting these spatial orderings was a major challenge. The Women's Feature Service, which in 1994 provided information on the preparations undertaken by the UN region “Europe and North America” for the Fourth UN World Conference on Women in Beijing/Huairou, placed Eastern Europe, Western Europe, Israel, Canada, and the United States in one region. Also included were Russia, geographically partly in Europe and partly in Asia, as well as Uzbekistan and Kyrgyzstan, geographically in Asia. The editorial team was well aware of how diverse the region was and was nevertheless willing to accept the UN spatial order. A text entitled “Who Are We?” which cites the appeal by the 1994 UN regional preparatory conference in Vienna “Women in a changing world—call for action from a regional perspective,” points out the contrived nature of the region. They write: “But how is a ‘regional perspective' possible in a collection of states so protean that it includes Andorra, Iceland, Moldova, Switzerland, Malta, Slovenia and—Canada? Not to mention France, Uzbekistan, Norway and the United States. Where is our common ground? In 5 days from now we will know the answer” (Women's Feature Service, 1994, p. 8).

The regional spatial order was also reflected in the NGO Caucuses at the parallel NGO forum of the Fourth World Conference on Women in Huairou, a suburb of Beijing. For example, regional divisions determined the structuring of the civil society campus and its Caucuses. Each official United Nations region had a tent of its own for meetings between organizations. Delegates from the post-socialist countries were largely assigned to the European region.

### Eastern Europe as a “Non-region”

As we will be seeing, integration of the subject category woman in a regional, scalar order had both advantages and disadvantages for women's rights activists from Poland. The advantage lay in the subordination of countries to the higher scale of the region in the spatial order defined by the UN. Many post-socialist countries in particular, at a time when the political “Eastern Bloc” had just collapsed and the Soviet Union disintegrated, had no immediate interest in acting as a common region. They were intent solely on gaining the recognition of the international community in order to assert their newly won sovereignty.

Neither among the states that had emerged from the former so-called “Eastern Bloc” nor among feminist actors who often felt themselves to be in political opposition to the old system, was a common “identity” really wanted. In my 2010 interview with the co-founder of Karat Coalition Kinga Lohmann, conducted in Warsaw, I learned that women from the former Eastern Bloc initially even had to overcome “distrust.” The difficulty of finding a stance on a common “identity” or organization is shown, for instance, by the interviews I have conducted, as well as by texts produced by women's rights activists. However, unlike the states of post-socialist Europe, women's rights activists had decided to accept the superordinate scale of region in order to gain a voice. Relations with Russia and with actors from Russia, in particular, are still often fraught with prejudice and mistrust, even where representatives of women's organizations are concerned.

The disadvantage of regional positioning is that scalar integration of the former political Eastern Europe in a common Europe means in practice subordination to Western Europe. To this day, Europe is largely equated with the European Union. But in 1995, Poland and the other Eastern and Central European countries were not yet members of the EU. At the same time, the post-socialist countries on the border between Europe and Asia are either marginalized or transferred to the Asian region. The relation of “Eastern Europe” to “Western Europe” was (and by some still is) defined as a “transition,”^23^ a “becoming Europe.” The relationship between candidates for EU membership and members of the Union was and has remained that between teacher and pupil (compare Kuus, 2007).

As Doreen Massey has shown in her study on the construction of North-South relations (Massey, 1999), the spatial difference is produced as a temporal difference. Such a relationship means that the prototype is taken to represent the whole and hence also the units subordinate in scale. Such similar interpretations regarding Eastern Europe as an early or incomplete version of developed Western Europe (however setting out from a different dichotomy to become Western) have been called into question by scholars (e.g., Burawoy and Verdery, 1999; Kuus, 2004, 2007; Kovacevic, 2008; Tlostanova and Mignolo, 2012; Hann, 2002; Todorova, 2009)^24^.

Another relation that played a role in the Caucus, and which decided the relational positioning of Europe, was the dichotomy between the global North and the global South. Regulska and Grabowska postulate that “the Polish women's organization was confronted by a debate defined in advance in international feminism, which limited itself to a dialogue between the First and Third Worlds” (2008, p. 47). Such a dichotomy left no space for articulating the ambivalent experiences from a “second world” perspective (Suchland, 2011, p. 837). According to the activists I interviewed, these forms of spatial synthesis and spatial ordering mean that topics that failed to fit within the North/South dichotomy were simply not addressed^25^. For this reason, many actors from post-socialist countries found it difficult to identify with the scalar order. Scholars such as Jennifer Suchland and Magdalena Grabowska do recognize the second world as a “site of global struggles” and argue for posing “the question of the implications of the ambivalent postcolonial status of some Eastern European locations for transnational feminist theory and practice” (see Grabowska, 2012, p. 387; or similarly Suchland, 2011, p. 854). With this, these authors are actually responding to claims for autonomy and postulates of difference, as feminist actors from former European socialist countries also partially articulated at the UN Conference on Women in 1995 in Beijing, where they called for a revision of the applied geopolitical divisions, dichotomies, and frameworks.

At the NGO Caucus of the UN Conference in the Republic of China, an East-East Caucus was called for. Working sessions of the resulting Eastern European Caucus criticized the Platform of Action, which, among other things, described poverty as a “short-term consequence of transformation” (East-East European Caucus, 1995). The Statement raised concerns, because of the “decline of the status of women,” the increasing unemployment, violence, human trafficking, and the more than 20 armed conflicts, that forced many women to leave their homes. Next to this the prohibition or planned prohibition of abortion was defined as the “most pressing concern for women” in the region (Ibiden). The caucus then relocated from Huairou to Beijing (Nowicka, 1995). The East-East Caucus criticism was formulated in a “Statement from the Non-Region,” which the women's rights activist from Poland Wanda Nowicka presented to the Beijing plenary session of the UN conference (East-East European Caucus, 1995). Later on, the situation was explained by an organization that emerged partly in reaction of those experiences the UN (and that involved as well Wanda Nowicka) in the following way: “During the UN Conference on Women in 1995 in Beijing, the CEE^26^ region was referred to as a ‘Non-Region,’ and today it continues to be situated somewhere between developing and developed countries. Not fitting perfectly well into the Global North or South framework resulted in the marginalization of our issues in international forums” (ASTRA, 2012, p. 1).

To strengthen its legitimacy and validity, the authors of the statement claimed to represent 400 women from 80 NGOs in 19 countries. An actor from Russia, Anastasia Posadskaya-Vanderbeck, wrote of the work done in the East-East European Caucus: “Those of us in the post-Communist countries know how the legacy of the Soviet empire prevents cooperation and communication among people of the region, so we considered it a special challenge to work out our own unified statement” (Posadskaya-Vanderbeck, 1995). The Statement from the Non-Region could be interpreted as an attempt to oppose a regional order. However, it did not really escape thinking in regional, scalar syntheses, for a statement by a region that claims to be a “non-region” is contradictory. This contradiction could be attributed to, among other things, the fact that subject positioning, the ordering in a spatial synthesis of a post-socialist Eastern and Central Europe, was also questionable for representatives of NGOs and women's rights activists. In the Statement from the Non-Region, we read: “Our group of countries is a non-region because there is no recognizable political or geographic definition for the region composed of countries in Central and Eastern Europe and the former Soviet Union” (East-East European Caucus, 1995).

A participant in the conference had this to say: “We were nobody, we were not identified, nobody, nobody knew at that time who we are, even we didn't know who we are, because it was chaotic times in a way and even structures like the UN were not sure how they should treat us, they did not know the differences of course. Eastern Europe was all in one basket”^27^. The question whether post-socialist Central- and Eastern Europe and Central Asia be at all named that way and if they form joint region or not or if there are any present common denominators (beyond the socialist past) has become an ongoing issue of debate and dispute, not only among activists, but also scholars until today^28^.

### Empowerment by Regional Representation: The Founding of the Karat Coalition and ASTRA Network

In political practice at United Nations meetings, subject constitution (“identity attribution”) is tied not only to the subjects themselves and their relations or the direct, social context but also to an intrinsic spatial dimension, because subjects are attached to and integrated in concrete notions of space and territory, for example politico-geographical entities. The possibility of attaining representation in the United Nations was therefore strongly dependent on regional classification. In an application for a project called “Regional action of Karat Coalition,” the authors wrote: “After arriving in Beijing we realized how important attachment to a specific region is” (Karat Coalition for Regional Action, 1999, p. 2).

The prospect of gaining leverage (also vis-à-vis individual states and their representatives) associated with regional subject constitution induced members of SKOP—‘95, on their return to Poland, to set up a regional Eastern and Central European organization for gender democracy, the Karat Coalition^29^, which was to have a watchdog function vis-à-vis states^30^. The idea of founding an independent regional organization grew in the course of interaction and experience at the UN World Conference on Women in Beijing in 1995 (compare e.g., Fuchs, 2003; Lang, 2009). Some of the Eastern and Central European NGOs represented there joined the coalition. The strategy of the Karat Coalition is summed up in a leaflet issued out by the organization by the following quotation:

“WE ARE A GATEWAY AND A MEGAPHONE. Through Karat, the voices of our member organizations are amplified and channeled together in order to be heard and listened to on a European level and beyond. As the only network of this kind in the [CEE] region, the important and difficult efforts of our members are internationally recognized when often undervalued in their own countries.”

Karat Coalition (n.d.)

Their aim, as stated in the project proposal for Karat Coalition, was, among other things, “to make our region visible at international fora” and “to represent our region and advocate for it in international fora“(Karat Coalition for Regional Action, 1999, p. 1). In the early years, Karat developed its own structures and a regional network. A highlight for Karat, whose work in UN processes (e.g., at meetings of the Commission on the Status of Women (CSW) and the quinquennial reviews of implementation of the Beijing Platform for Action for the UN regions) was the presentation of a first joint regional report at the 43rd meeting of the UN Commission on the Status of Women in New York in 1999 (Karat Coalition for Regional Action, 1999). In the course of time, the Karat Coalition attained the status of a regional voice at the level of the United Nations through its regular presence. It had itself become a representative organization and had come to be recognized as such. The strategy of the Karat Coalition for attaining representative status had proved successful. This is demonstrated by the fact that the Karat Coalition has been called upon by international organizations such as the United Nations to report on the situation in the region or in the individual countries of the region, or even to represent the civil society of the region and specific countries of the region. Karat Coalition also holds a status of ECOSOC at the UN Social and Economic Council.

At presentations of the regional Karat Coalition at international fora's such as organized by UN or the EU Institutions, examples from member countries are repeatedly cited and representatives from the relevant countries attend by invitation. Member organizations see membership in a regional coalition above all as strengthening their position in negotiations with government representatives. Transnational action can thus further the strategic aim of enhancing an organization's standing at the national level (compare Keck and Sikkink, 1998, 1999, p. 69).

For strategic reasons, and owing to similar experience with a lack of profile and voice, Wanda Nowicka (co-author of the Statement from the Non-Region, important advocate in the international arena, and a key figure in the women's rights NGO landscape in Poland)^31^ also co-founded the regional network ASTRA in 1999. In this case, the principle of regional spatial ordering was applied to a thematic NGO focusing on reproductive rights. One important motive for establishing the organization as a regional body was growing regionalization at the political level.

The examples show that scale can be used strategically to attain legitimation and representation, as well as symbolic dominance over units subordinate in scale. A scalar practice is illustrated by the cartographic presentation of the Karat Coalition on the organization's website (website-header of Karat Coalition, accessed in October, 2010).

The Karat region includes post-socialist countries (including Germany^32^), Eastern Central Europe, and the former Soviet Union. The map does not show the exact location of the individual member organizations (this would give a quite different, selective picture focused on cities), but a region-wide presentation that includes national borders. This shows the coalition as powerful and extending far beyond individual states.

The different countries in the region belonging to the coalition can be accessed from the website. Although the map emphasizes Poland, it is only a scalar part of a large, regional Karat unit. Actors from Poland occupy leading positions in the regional initiatives mentioned. Three of the most important gender-democracy advocacy organizations that address post-socialist Eastern Central Europe and to some extent Asia have their official headquarters and secretariats in Poland: the Karat Coalition (Warsaw), ASTRA (Warsaw), and the Network of East-West Women (Gdańsk).

The interviews conducted with actors from the Karat Coalition and ASTRA show that they are aware of the contrived nature of regional spatial syntheses. The “identity” and composition of the region is constantly being rethought within the organization. In the case of the NGOs under study, such as the Karat Coalition, this process is also one of negotiating changes in the external boundaries and membership of the organizations, as well as in the shifting definitions of the region, which serve to legitimize their own spatial synthesis.

Whereas, the 1995 Statement from the Non-Region had still seen transition as a common characteristic, the program of the Karat Coalition as well as members of the secretariat, many years after the EU accession of the states of some its members, see a change of integration in a spatial construction through the relation of development aid. The concept of development thus replaces the concept of transition which, in the 1990s, had defined what the countries of post-socialist Europe had in common in the UN context (to quote the Statement from the Non-Region: “We are bound by the common problems associated with the transition to democracy,” and the region is termed “Countries in Transition in the ECE Region”). The difference, however, is that, in the eyes of NGO actors from the new EU member states like Poland, as well as the NGOs from this region, these countries after EU-accession have to assume the role of “prototype,” of “mediator” or “bridge”. Such self-positioning as mediators between the “East” and “West” is (or at least had been until the democratic crisis that ensued with the far right nationalist takeover after the elections in 2015) very popular among intellectuals, and politicians in Poland^33^. From the perspective of the Karat Coalition, the inclusion of outlying neighboring regions into the political project of “Western European” type of democracy is an important task.

The status of the former socialist countries changed after the EU enlargement to the East. EU member states were regarded as secure and democratic—a perception that partially changed after the nationalist takeover and the partial dismantling of democratic governance structures in Hungary and Poland^34^.

The changing position of Central Europe after the EU accessions of 2004 was described by Alan Dingsdale as “shifting from a status as the Western edge of the East, to being, once again, the Eastern edge of the West” (2002, p. 267). In the cartographic self-presentation of the Karat Coalition, too, Poland is located relationally within the scalar unit “Western.”

Relational integration in a common region is expected to allow organizations from the post-socialist, new donor countries of the EU to pass on their experience with “transition” to member organizations of the Karat Coalition outside the EU (and particularly from countries in the Commonwealth of Independent States, CIS). A fear that has been expressed in interviews was that, for example, states from the CIS region and Central Asia would distance themselves from the democratic principles espoused by members of the European Union and from women's human rights. Collaboration is seen as a guarantee that access to “Europe” is not lost for non-EU members. The expertise in former recipient countries from the Karat Coalition that have become EU-members is used to influence development aid policy in the new donor countries and to strengthen the gender component in programs. This spatial synthesis of the Karat Coalition, which integrates both countries receiving development aid and donor countries, also gives access to development aid funding. Regional divisions as practiced by donors (private foundations, government funds, and international organizations) mean in practical terms that funding is also distributed along regional lines. But the NGOs that practice other spatial syntheses do not often fit into the distribution pattern. The lack of funding sources makes it difficult to maintain the cohesion of organizations. For, in the given socio-political context, there are few possibilities to carry out projects in the self-defined “region” without external funding. On the other hand, introducing the subject of gender-democratic development lends new legitimation to the Karat regional spatial synthesis in a situation in which the post-socialist region is becoming increasingly differentiated and the importance of the common political past (membership in the state-socialist community of states) is declining.

However, members of Karat criticize in interviews the official development policy of governments and international organizations and call to mind their experience with incapacitation and the risk of reproduction. The term of “development” is partly replaced by the concept of “cooperation.”

This brings us back to Doreen Massey's criticism of the hegemonic distribution of space through temporal difference, where one spatial construction is understood as being equivalent to another, related space construction, albeit at an interval of time, and with a development path being incorporated in the definition of space (Massey, 1999). Scalar, relational integration that corresponds to the idea of a development path can thus also lead to incapacitation.

Looking back on their experience, Karat members have also come to criticize the development aid policy of the 1990s, which influenced the transformation of post-socialist countries as a means for automatically transferring concepts on the development agenda. As one representative of a Karat Coalition member organization and UN consultant said in an interview: “Especially the USA, I have to say, made a lot of mistakes; they tried to enforce their vision of society on countries where this concept was completely alien. So after all the years I've been in development, I'm pretty sure you simply cannot, you cannot, without any kind of modesty, when you have maximal respect for society, even if it's a poor society, you cannot change the world to suit yourself, in your own image”^35^.

Members of the Karat Coalition are aware of limits to such scalar strategies based on hegemonic spatial regimes/orders (be it in resistance to or in mirroring existing regimes). Such a reflection on the structures of belonging is also expressed in a quote from an interview with a representative of a Karat Coalition member organization from outside of Poland: “Karat has to perhaps find a way not to think about territorial division, but instead start to think how the network could work regardless of divisions of regions. The Karat Coalition is an NGO, we don't need to follow the structure, to be a servant of the structure. I think there is a huge opportunity for the Karat Coalition to broach new themes, because we can be much more flexible, to open up to new things which are emerging now.” Some members of the Karat Coalition have accordingly expressed the wish to practice other forms of spatial synthesis with regard to the organization. They demand that the coalition be more flexible and action-oriented in its own spatial synthesis.

Over the years, Karat's strategies have been developed further also in terms of the strategies applied and issues addressed. The institutionalized regional structure however proved to have a powerful impact, also implicitly influencing the “civil society” (e.g., by political opportunity structures or funding and networking opportunities such as national development aid, the EU neighborhood policy). In the long run, the change in the framework of belonging (many years after the EU integration of some former state-socialist countries) made it very difficult for Karat to continue its own scalar agenda. On top of that, a right wing, nationalist, and fundamentalist religious backlash forced NGO's (including Karat member organizations) in many countries to focus on national issues while lacking human and economic resources to engage regionally as well. NGOs specializing in advocacy depend on funding in order to carry out their work. It requires specialist skills and a profound knowledge of institutional mechanisms, as well as mobility and continuity in order to follow up decision making processes. This accounts especially for transnational advocacy. The backlash itself has been considered as an international problem that is not specific only for Karat coalition's region (post-state socialist Europe and Central Asia). It requires action beyond an east/west divide. After a general assembly conducted electronically in December 2018, Karat decided on its dissolution as a regional network. The final dissolution of Karat Coalition indicates how difficult it is to maintain an alternative spatial synthesis, especially when that synthesis is not supported or compatible with dominant and formalized spatial regimes of belonging, and the opportunity structures they provide.

## Recent Developments. Turning Human Rights Scales Upside Down

Why is it important to refer today to the events that accompanied the UN *World Conference* on Women and the struggles over gender regimes in *1995*?

Despite the immense critique of the UN Human Rights framework and its language, including on the part of feminist actors for its being far behind the demands of feminist social movements^36^, the introduction of the notion of gender and the definition of women's rights as human rights, as a norm and point of reference for critique, has major relevance. The simple idea that women be granted autonomy in relationship to the family is still highly contested today, and human rights discourse still remains an import reference and instrument for social movements.

Although the UN is weakened and the European Union has become much more important when it comes to political frameworks, both outside of Europe and within the new EU member or candidate countries, the UN Human Rights framework continues to be important because it is used not only for setting and legitimating norms internationally but also within political struggles between various social groups, state and non-state actors, including social movements, NGOs, and religious organizations.

Human Rights remains a point of reference for various actors (in both negative and positive ways), when it comes to such issues as protection from violence within the family or issues of reproduction, to take an example from women's rights.

At heart, many of the struggles that we can observe today and that involve the religious and conservative (far) right are struggles over regimes of belonging, largely guided by attempts to restore a so-called “natural order”^37^. The neoconservative backlash that is taking place in many countries (including Poland), and that is often accompanied by a revival of ethno-nationalism, seeks to subordinate individual human beings not only under the *nation* but under the *family* as well. This backlash does not stop on national grounds. It is played out at the UN, where diverse actors work at fundamentally transforming the human rights framework. The efforts include attempts to change regimes of belonging on the level of nation states, the UN, and the European Union, in order to establish a familist regime. International and supranational organizations are meant in future to be used as instruments for the top-down implementation of such changes on national grounds. A first victory in this effort is the Resolution of the Human Rights Council “On the Protection of the Family” from 2014. This resolution was made possible due to pressure from, among others, states and NGOs, including coalitions of Christian (Vatican, Russian Orthodox Church) and Muslim religious and state actors, for instance the United Nations Family Rights Caucus at the CSW61 2017 in New York. In the joint declaration, the Caucus declared that their mission is to “protect and promote the natural family as the fundamental unit of society as called for in Article 16 of the UN Declaration of Human Rights.” Furthermore, they declare: “The one thing that unites us all, regardless of faith, national origin, or cultural background is our understanding that a nation's strength and the well-being of its people depends largely on the stability and strength of its families” (UN-Family Rights Caucus, 2017). That the familist ideology is often closely interrelated with nationalism can be traced, for example, in the activities of the UN lobbyist NGO called Family Watch International (FWI), where demands of “protecting the family” are linked with claims for “protecting national sovereignty” (Family Watch International, 2017). As women's rights advocates working for AWID (Association for Women's Rights in Development) have observed, this implies a major shift from the protection of individuals to the protection of institutions and social regimes (AWID and OURs, 2017). This would imply that the idea of rights granted to individuals in order to protect them from violence experienced at the hands of states and communities is moved toward the idea of protecting scalar constructs of belonging. In this way, the basic idea of human rights protecting individual humans from powerful collectives and institutions, such as states, religious communities, or the family, is turned upside down and envisioned as a tool for those who already govern communities. In other words, the idea that rights are granted to individuals in order to protect them from violence at the hands of states and communities is displaced by another idea that seeks to protect scalar constructs of belonging. However, changing a UN framework would be a very long, step-by-step, and multi-leveled process. How this struggle over regimes of belonging will end is still an open question, and it's apparent that this process will not develop uniformly within all UN bodies. While the UN resolution “On Protecting the Family” has been confirmed and further developed through two other resolutions in 2016 and 2017, UN frameworks and bodies by and large still follow up on the agenda of “Women's Human Rights,” as it was confirmed and developed in the Beijing Declaration and Platform for Action produced thanks to the Fourth World Conference on Women in September 1995. Sexual and reproductive health and the rights of women and girls are still central in the human rights framework. The concluding observations of the United Nations Human Rights Committee on the periodic report under ICCPR (International Covenant on Civil and Political Rights) of Poland from November 2016, exemplify this. The Committee expressed concerns about the high number of women victims of domestic violence and urged the Polish state to “refrain from adopting any legislative reform that would amount to a retrogression of already restrictive legislation on women's access to safe legal abortion” (Human Rights Committee, 2016).

## Conclusion

In this article, I have discussed the importance of scales such as “Europe” and the “family” in women's rights advocacy, their impact on the possibility of representing “Polish” and “European” women at the Fourth UN World Conference on Women and NGO Caucus in Beijing in 1995 and, above all, the impact of scale on political agency.

Instead of taking an identity approach and discussing supposed “differences” between women positioned as “Eastern” or “Western European,” which would lead feminist actors to demand the recognition of their difference, I have focused on the structural reasons that political agency has become constricted and on strategies that aim at changing those very structures, such as institutionalized regimes of belonging.

Keeping in mind that scalar regimes are not only constructs but, when institutionalized, they become very influential in organizing not only daily life but politics as well, it is not simply possible to ignore them. Political action also requires dealing with pre-existing structures of belonging, such as the organization of categories of humans within hierarchical scales and one's own positioning within them. The very form scalar regimes of belonging take; the hierarchical distribution of identities within lies at the very foundation of many socio-political struggles. Those struggles always take place in a specific context, in a specific spatial order, and from a specific position. As the example of positioning by women's rights actors from Poland at the Fourth UN World Conference on Women has shown, the possibility of advancing political demands and of attaining representation depend strongly on the positioning of the actors and movements involved in a spatial order of belonging.

The very positioning of identity categories within regimes of belonging lies at the very foundation of many socio-political struggles; The question of power that is implicit in these positionings is especially crucial in the conflict between the national-Catholic and gender-democratic camps in Poland.

I have argued that the UN spatial order, which granted autonomy to the category “women” in regard to the category “family,” allowed gender-democratic actors from Poland to gain political agency. The spatial order of belonging gave them some advantage in their struggle against religious activist women from anti-choice Catholic family organizations, in regard to the question of the legitimate representation of “Polish women.” However, the activists from Poland who are struggling for gender democracy also experience some disadvantage due to the superiority of a regional spatial division and their symbolic and formal inclusion into Europe, which is represented by core countries of the former “West.”

The construction of autonomous regional spatial syntheses and their perpetuation in the form of organizations was closely associated with the United Nations, the development programs [regional until 2004 [CIS-CEE]], the 1995 UN World Conference on Women, and the Beijing Process, since these events and the associated consultation procedures were organized on a regional basis. The organizations mentioned use regional categorization, spatial “we-production,” strategically to gain representation in a spatial ordering that favors regional constructions over other constructions and subordinates the national space to the greater entity of the region.

Karat Coalition's dissolution in 2018, after more than 20 years, indicates that for advocacy NGOs networks maintaining an own scalar synthesis (that does not follow institutional regional patterns) is difficult in the long term. Furthermore the UN, which had a consolidating impact on gender democratic movements from CEE in 90s, lost its overall significance as a forum to enhance women's rights on national levels^38^. What this means for transnational activism requires further observation and research. Recent developments indicate a decline of transnational advocacy work aiming at transforming existing institutions (e.g., the UN, station states), because opportunity structures are shrinking. Simultaneously, contentious politics and new social movements are rising locally, which also network and spread beyond nation states.

The strategies of women's rights activists are based on a scalar hierarchy. The national principle in spatial ordering is not canceled or transcended but integrated in the spatial construction, as the Statement from the Non-Region, the East-East Caucus, and the regional Karat Coalition show.

What in the above-discussed cases is decisive in questions of representation and the ability to perform political agency is not so much the difference between same and other but that between universal and particular, as well as the scalar time-space hierarchy of prototype and becoming. This concerns both the positioning of the category woman (as autonomous or integrated scalarly in the family), as well as the positioning as a region, whether as Europe or as an autonomous, post-state socialist European region. It is exemplified in the entrenchment of development paths, such as *becoming* family, *becoming* Europe. The examples I have given show that inclusion, too, in the sense of scalar integration within hierarchically organized regimes of belonging, can contribute to restricting access to representation and to spokesperson functions.

Inclusion, therefore, might effectively limit political agency and the ability to influence discourses and policies. Thus, struggles, such as those observed at the UN World Conference on Women in 1995, cannot be adequately described using only the limited framework of “identity” and “recognition.” They were first and foremost struggles over the composition of regimes of belonging and their internal hierarchies. The scale in those hierarchies of belonging draws boundaries around social categories and this has an impact on the relative autonomy and agency of a category. It organizes political exclusion through inclusion.

## Data Availability

The datasets for this manuscript are not publicly available because they might contain confidential information. Requests to access the datasets should be directed to JB, ramme@europa-uni.de.

## Ethics Statement

This study was carried out with informed consent from all subjects. All quotes used in this article have been authorized prior to publishing by the interviewed persons and their written informed consent has been obtained. Ethics approval was not required at the time the study was conducted as per Institution's guidelines and national regulation.

## Author Contributions

The author confirms being the sole contributor of this work. She was responsible for conducting the research, archival research, participatory observation, conducting interviews, the analysis of empirical material, the framing of the paper, the writing, and the literature review. The author has approved it for publication.

### Conflict of Interest Statement

The author declares that the research was conducted in the absence of any commercial or financial relationships that could be construed as a potential conflict of interest.

## Footnotes

^1^They include conferences, meetings, consultations bringing together representatives of NGOs, governments, and international organizations, and meetings in the context of these processes that assemble NGO representatives and so called “civil society” actors. The term civil society here refers to an actor categorization and intermediary space between society and states or international organizations that has relevance within UN-processes. The term itself is controversially discussed (more recently for the polish context see for example Fábián and Korolczuk, 2017).

^2^Some of the interviewees lived abroad, but their activities and organizations focused on political and societal changes in Poland or they returned to Poland after system change.

^3^I refer here to the definition of familism of Notz (2015, p. 17–19).

^4^My initial research focused on processes of “transnationalization” and existing transnational activism, especially I aimed at finding out the reasons for establishing regional post-state socialist “Eastern European” initiatives. My research also focused on the problem of “East-West divisions” within feminism and possible differences between “regions”. I collected a very broad basis of empirical data. The development of an adequate theoretical approach emerged throughout the study and the ongoing analysis of data. This approach to the empirical material allowed me to recognize problems and patterns throughout the research. Based on these emerging patterns samples and particular exemplary cases for were selected and analyzed more in-depth within the study. From all of the interviews for this particular sub-study I focused on initiatives and actors that have been involved in UN-processes and that have a “Polish” origin. The information from the interviews was compared with other interviews, documents from UN-Archives and archives of the organizations under study, information coming from academic publications, official publications and movies documenting the participation of feminist activists at the UN.

^5^Above all the archives of the Women's Center PSF (PSF Centrum Kobiet) in Warsaw (dissolved), the document collection of the Karat Coalition, the archive of NEWW network in Gdansk, as well as documents and publications made available by participating actors. Next to this I have used materials collected in my private archives since the early 90s.

^6^In the 90s in Poland there have been a limited number of feminist NGOs and advocacy actors. Therefore it was possible to interview representatives of most NGOs and most individual actors from Poland that advocate for women's right also on international fora. Qualitative in-depth interviews have been conducted with a large number of gender-democratic NGO and individual actors (individuals were mainly specialists for advocating at the UN and EU) from Poland, Central-Eastern Europe, Central Asia and the U.S.A. Next to this I included all transnational networks that are based in Poland and aim at influencing policy and have formalized structures. I was also searching for other border-crossing networks including members of post-state socialist countries.-Interviews have been conducted by the author in the years 2009-2013. The information from the interviews was compared with other interviews, documents from UN-Archives and archives of the organizations under study, information coming from academic publications, the press, official publications and movies documenting the participation of feminist activists at the UN. They provided background information helping to understand the processes analyzed in this article. Quotes from interviews with individual members of those organizations have been authorized (written consent) and do not represent the opinions of the organizations in general. My initial broad research on gender-democratic activism transcending borders revealed limits of the existing theoretical and methodological approaches on transnational activism and struggles for recognition that was focusing very much on the problem of “recognizing” identities and the legitimacy of claims postulating a difference.

^7^Among other things, I have taken part in various events, conferences, and important internal meetings of the organizations, such as board meetings and workshops of the Karat Coalition on, for instance, development and UN human rights, the general meeting of the Karat Coalition in Bucharest in 2010, and the 54th meeting of the CSW UN 2010 thanks to NEWW. The participant observation at the 54th meeting of the CSW UN United Nations in 2010 was crucial for me in order to realize the importance of scalar socio-spatial arrangements in organizing subject-positions and its influence on political agency, as well as, above all, the difficult position of those who do not fit into pre-established regimes of belonging. Also I have been taking part at parallel events and caucuses organized during the CSW61 in New York in 2017. Those practical experiences provided important practical knowledge for comparison and the reconstruction of the events and advocacy work described in the interviews. And last but not least I have been analyzing the activities, discourse and strategies of the most vocal opponents of gender equality in the public discourse in Poland.

^8^I would like to thank John Binnie for reviewing an early version of my paper (2010) from the perspective of human geography and his very helpful suggestions on the criticism of scales.

^9^Shadow reports” are alternatives to governmental reports. In this case the shadow report provided a “civil society” perspective on the situation in Poland that provides alternative information to the official report of the Polish Government.

^10^Statements in interviews with Solik, Aleksandra (member of SKOP and Karat secretary, former member of the NGO Neutrum, Federation for Women and Family Planning and the ASTRA-Network), interviewed by author in 2009 and 2010 in Warsaw. Interviews with Chołuj, Bożena (member of SKOP and individual member of Karat, Head of Gender Studies at Warsaw University) interview by author in 2009 and 2010 in Warsaw.

^11^Interviews conducted among others with B. Chołuj in 2009 and 2010 in Warsaw by author.

^12^Interviews conducted with A. Solik in 2009 and 2010 in Warsaw by author. Revision in 2019.

^13^For a discussing of the relationship between the Polish State and Catholic Church see among other the works of Ramet (2014, 2017), Ramet and Borowik (2017) or Frances Millard (1997). For the particular church stances on gender and reproductive issues the studies by Mishtal (2009, 2015), Szwed (2015), Szwed and Zielińska (2017) and Kulawik (2017a,b) are giving important insights. In regard to the bodily dimension of citizenship and the struggle for autonomy see e.g. Outshoorn et al. (2012).

^14^After an interval of time, the office was re-established in 1994 shortly before the Fourth UN World Conference on Women. See also court rulings in the database Internetowy System Aktów Prawnych [Internetowy System Aktów Prawnych (ISAP), n.d.] on the function of the Pełnomocnik Rzaa̧du do Spraw Rodziny i Kobiet.

^15^On the changes in name until the end of the 1990s see Nowakowska, Urszula “Instrumenty państwa na rzecz awansu kobiet” (Nowakowska, 2000).

^16^In the mid-90s such as the Christian National Union (Zjednoczenie Chrześcijańsko-Narodowe, ZchN), Electoral Action Solidarność (Akcja Wyborcza Solidarność, AWS), and later the League of Polish Families (Liga Polskich Rodzin, LPR), as well as the still existing Law and Justice (Prawo i Sprawiedliwość, PiS).

^17^For a discussion of human rights law and the relationship to religious norms, gender equality and the family see also e.g., Diduck and Raday (2012).

^18^The *Convention on the Elimination of Discrimination against Women* (CEDAW) was adopted by the UN General Assembly in 1979. The United Nations Committee on the Elimination of Discrimination against Women (CEDAW) requires members of the convention to submit a report every four years, which is then examined by the committee (UN Women (n.d.)). For a discussion about the impact and critic of CEDAW see e.g., Raday (2012b).

^19^The history of feminist struggles to influence international human rights norms and the impact of UN in terms of enhancing the situation of women and feminist transnational activism around the UN has been subject of research especially in the field of political science, international relations, sociology and history (for an overview see e.g. Keck and Sikkink, 1998; Ruppert, 2002; Friedmann, 2003; Joachim, 2007; Krook and True, 2012).

^20^United Nations Economic Commission for Europe (UNECE), United Nations Economic and Social Commission for Asia and the Pacific (UNESCAP), Economic Commission for Latin America (ECLAC), United Nations Economic Commission for Africa (ECA) and United Nations Economic and Social Commission for Western Asia (UNESCWA). See UN Regional Commissions (RCNYO (n.d.)).

^21^However, not all these criteria have to be met. For example, the European Union, the United States, Canada, and Israel are all assigned to one region. Obviously, it does not meet any geographical criteria. The situation is similar with other United Nations regions. For example, Latin America and the Caribbean is a region defined in terms of “historical, economic and cultural ties” (ECLAC, 2013).

^22^This is particularly true of former European colonial powers, but also, for example, of the United States.

^23^Between 2009 and 2010, the time when the interviews were conducted, actors feminist NGOs are already distancing themselves from the concept of “transition.” The concept “transition” is now no longer used so frequently in the feminist movement. Also academic feminist scholars critically discussed the term “transition” (e.g., Gal and Kligman, 2000; Einhorn, 2006).

^24^The sometimes problematic dimensions and relationships within transnational feminism and East/West divisions (e.g., Funk, 1993, 2006, 2007; Chołuj and Neusüß, 2004b; Frunză and Văcărescu, 2004; Muharska, 2005; Lukić et al., 2006; Slavova, 2006; Regulska and Grabowska, 2008; Fábián, 2014) and the marginalization of the so-called “second world” within theory and feminist practice have again become in recent years of major interest to gender and sexuality studies scholars. Scholars also draw on approaches borrowed from postcolonial theory (e.g., Kulpa and Mizielinska, 2011; Suchland, 2011; Grabowska, 2012; de Haan et al., 2013; Koobak and Marling, 2014; Tlostanova, 2015; Tlostanova et al., 2016; Mitić, 2017). Next to this, a growing body of literature critically discusses the link of “europeization” and neoliberal transformation (e.g., Koobak and Marling, 2014). Whereby, some state that feminism post-state socialist countries in Europe was “designed” by foreign agencies and foster neoliberalism with the help of human rights framework (e.g., see Ghodsee, 2004; Gregor and Grzebalska, 2016). The above mentioned critique, however, are first of all political and philosophical interventions that address many important problems (e.g., class based exploitation, ngoization etc.), but simultaneously miss out the complexity of both, the feminisms in the post-state socialist region and the advocacy work aiming at the transformations of institutions and structures. From my empirical research I learned that the feminist movement in Poland was highly diverse from the early 90s on (e.g., including grassroots feminisms that were opposed to capitalism. Feminists working in advocacy as well have been critically addressing the negative impacts the “transition” to a marked based economy (such as the describe statement of non-region). For a further critique of the “imperial critique”/“feminism-by-design” thesis see among others Funk, 2006; Ostertagová, 2016).

^25^Interviews with Lohmann, Kinga. (SKOP, Co-founder and Executive Director of Karat Coalition). Interview by author in 2009, and 2010 in Warsaw. Interview with W.N. Interviewed by author in 2009, Warsaw.

^26^CEE stands for Central and Eastern Europe.

^27^Karat Coalition member (N.N.), participant in the conference in Huairou 1995, interview conducted by the author in Warsaw in 2010.

^28^Not only among feminist activists and NGOs, but also among academics there exist a variety of opinions and analysis on whether we can distinct a center-“Eastern European” (based on the east-west dichotomy), “Center-Eastern European”, Center-Eastern European and Central Asian” region. While some scholars referred to the socialist legacy in order to explain further developments in the post-state socialist countries, others argue that there exist no common characteristics, or that some developments are common for so called “east” and “west” countries. For example Roobak and Marling, who draw analogies between post-socialism and post-colonialism, next to this, write that “CEE is not on a different temporal plane, but facing the same challenges from neoliberal ideologies as transnational feminisms. Our focus should therefore be on co-presences, interactions and interlocking understandings and practices rather than divisive dichotomies” (2017, p. 339–340).

^29^Karat is the name of the Warsaw hotel where the founding meeting of the organization took place in 1997. For more information on the Karat Coalition see as well Marksová-Tominová work (Marksová-Tominová, 2006).

^30^Interviews with B. Chołuj conducted in 2009, 2010 in Warsaw and by the author.

^31^A former member of Neutrum, SKOP – ‘9 and the Federation for Women and Families. She was also a member of parliament (Ruch Palikota) and marshal of the Sejm (speaker of the lower house of parliament) in the seventh legislative period.

^32^However, the participation of Germany is more symbolic in nature. Actors living in Germany occupy seats on the supervisory board. There are no member organizations in Germany.

^33^However, in other contexts the bridge metaphor often also indicates that Poland is distinct from the “East” and assumes for it a position of “Western” superiority. This ambivalence reflects the semiperipheral position of Poland and also that of the other former state-socialist countries within “Europe” (compare Boatcă, 2006).

^34^In the German Media for example Hungary and Poland were described as “model pupils” (“Musterschüler”) that have turned into “problem-children” (“Problemkind”) (e.g., Makro, 2016). In December 2017 the European Commission decided on activating the procedure of the Article 7 of the Treaty on European Union against Poland. In September 2018 it was activated against Hungary. The procedure may lead to suspend certain rights of breaching the EU's founding values European Union member states (European Parliament, 2018).

^35^Anonymous. Interview with representative of a non-Polish Central European Karat Coalition member organization. Interviewed by author in 2010 in Warsaw.

^36^A good summary of the feminist critique of the human rights discourse from the perspective of theory has been provided by Mullally (2006).

^37^“Natural order” is a popular phrase within neoconservative and far right discourses. One well known example is the brochure and manifesto “Restoring the Natural Order: an Agenda for Europe” (see also Datta, 2018).

^38^The view that the UN lost its impact is shared by many observers. According to interviews with advocacy actors (e.g., Interview with Lohmann conducted in 2019 by author) the space to advocate at the UN has dramatically sized down. I had similar observations during participatory observation at the CSW in 2017.
